# Fabry disease, respiratory symptoms, and airway limitation – a systematic review

**DOI:** 10.3402/ecrj.v2.26721

**Published:** 2015-06-26

**Authors:** Camilla Kara Svensson, Ulla Feldt-Rasmussen, Vibeke Backer

**Affiliations:** 1 Department of Respiratory Medicine, Bispebjerg Hospital, Copenhagen, Denmark; 2 Department of Endocrinology, Rigshospitalet, Copenhagen, Denmark

**Keywords:** Fabry disease, pulmonary involvement, symptoms, pulmonary function test

## Abstract

**Background:**

Fabry disease is an X-linked disorder caused by a deficiency of the lysosomal enzyme α-galactosidase A, resulting in accumulation of glycosphingolipids in multiple organs, primarily heart, kidneys, skin, CNS, and lungs.

**Materials and method:**

A systematic literature search was performed using the PubMed database, leading to a total number of 154 hits. Due to language restriction, this number was reduced to 135; 53 papers did not concern Fabry disease, 19 were either animal studies or gene therapy studies, and 36 papers did not have lung involvement in Fabry disease as a topic. The remaining 27 articles were relevant for this review.

**Results:**

The current literature concerning lung manifestations describes various respiratory symptoms such as dyspnoea or shortness of breath, wheezing, and dry cough. These symptoms are often related to cardiac involvement in Fabry disease as respiratory examinations are seldom performed. Pulmonary function tests primarily show obstructive airway limitation, but a few articles also report of patients with restrictive limitation and a mixture of both. No significant association has been found between smoking and the development of symptoms or spirometry abnormalities in patients with Fabry disease. Electron microscopy of lung biopsy and induced sputum show lamellar inclusion bodies (Zebra bodies) in the cytoplasm of cells in the airway wall. X-ray and CT scan have shown patchy ground-glass pulmonary infiltrations, fibrosis, and air trapping. Fibrosis diagnosed by high-resolution CT has not been significantly correlated with lung spirometry.

**Conclusion:**

Consistent findings have not been shown in the current literature. Pulmonary function tests and registration of symptoms showed various results; however, there is a trend towards obstructive airway limitation in patients with Fabry disease. Further studies are needed to evaluate pathogenesis, progression, and the effects of treatment.

Fabry disease is an X-linked disorder caused by a deficiency of the lysosomal enzyme α-galactosidase A. This deficiency causes a progressive accumulation of glycosphingolipids (primarily globotriaosylceramid) in multiple organs, resulting in decreased perfusion, cellular dysfunction, tissue remodelling, development of fibrosis and, consequently, organ damage (1).

The early manifestations of Fabry disease appear in childhood and are usually acroparesthesia, angiokeratomas, and hypohidrosis. Complications such as left ventricular hypertrophy (LVH), kidney failure, visual disturbances, hearing loss, tinnitus, cerebrovascular involvement, and gastrointestinal disturbances develop later in life (1, 2).

The general assumption has been that hemizygous men have more pronounced manifestations as well as earlier onset than found among heterozygous women, but newer theories suggest that women can be as severely affected as men. The most frequent mutations of the α-galactosidase A (*GLA*) gene are point mutations. The classic forms of Fabry disease are associated with mutations resulting in complete loss of function of the enzyme, whereas milder phenotypes and late variants might be associated with a substitution of a single amino acid (2, 3). The observed different phenotypes in women might also be explained by variable X-inactivation (4).

According to Danish guidelines, Fabry disease is diagnosed by 1) low activity of the enzyme α-galactosidase A in leukocytes and increased excretion of globotriasylceramid in urine and 2) detection of a mutation in the GLA gene. Apart from general supportive treatments such as angiotensin converting enzyme (ACE) inhibitor and angiotensin receptor blockers, painkillers, antidepressants, acetylsalicylic acid, clopidogrel or similar, the current treatment of Fabry disease is enzyme replacement therapy (ERT). ERT of patients with Fabry disease might limit the tissue damage and slow down the development of end-stage illness of the involved organs, but this treatment has only been possible for the last decade, and a large number of patients might already have been at or close to end-stage organ failure before treatment became available.

Involvement of the lungs in Fabry disease, and the severity of this, is still an issue of dispute, as very few publications exist on this subject. The hypothesis is that patients with Fabry disease develop interstitial lung diseases due to tissue remodelling in the alveolar surroundings causing fibrosis induced by accumulation of glycosphingolipids. However, if the remodelling includes the bronchial tree, one could hypothesise that chronic airway limitation might develop, resulting in the development of progressive airway obstruction.

In this review, the existing literature is compared and the pulmonary manifestations in Fabry disease elucidated and summarised.

## Materials and method

**Fig. 1:**
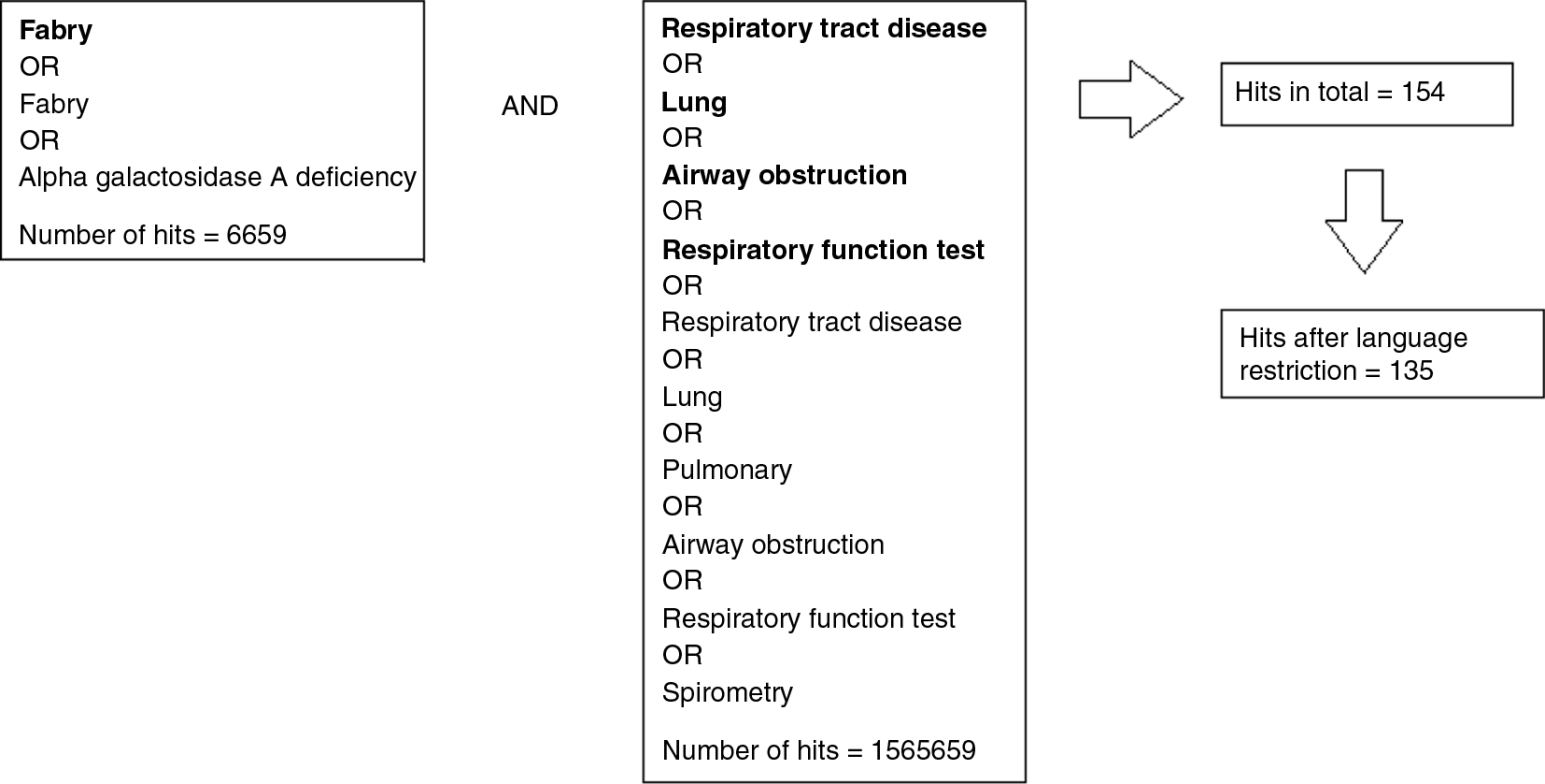
Illustration of the search profile. Words in bold letters are MeSH terms, whereas the others are words in text.

A systematic search was performed using the PubMed database on 2 July 2014, with the use of MeSH terms. To include literature to which MeSH terms have not yet been attached, words in text were also used in the literature search. Figure 1 demonstrates the search profile.

### Search words

#### Inclusion criteria

“Search (Fabry disease [MESH] OR Fabry OR alpha-galactosidase A deficiency) AND (“‘Respiratory Tract Diseases’” [Mesh] OR “‘Lung’” Mesh] OR “‘Airway Obstruction’” [Mesh] OR “‘Respiratory Function Tests’” [Mesh]) OR (Respiratory Tract Diseases OR Lung OR Pulmonary OR Airway Obstruction OR Respiratory Function Tests OR Spirometry)”. The search profile resulted in 154 articles.

#### Exclusion criteria

With a language restriction using only papers written in English, Danish, Swedish, and Norwegian, the result was reduced to 135 articles.

A total of 53 out of the 135 articles did not concern Fabry disease but were present because the authors were named Fabry, they mentioned a Fabry-perot filter, or they simply mentioned Fabry disease in a passing remark, and they were therefore excluded.

Additionally, 11 animal studies and 8 laboratory studies or gene therapy studies were excluded.

A total of 36 papers that did not have lung involvement in Fabry disease as a main topic (or did not mention it in abstracts) were excluded. These involved heart, kidney, craniofacial involvement, priapism, quality of life, anaesthesia, and the administration and availability of drugs in different countries.

#### Final material

**Fig. 2:**
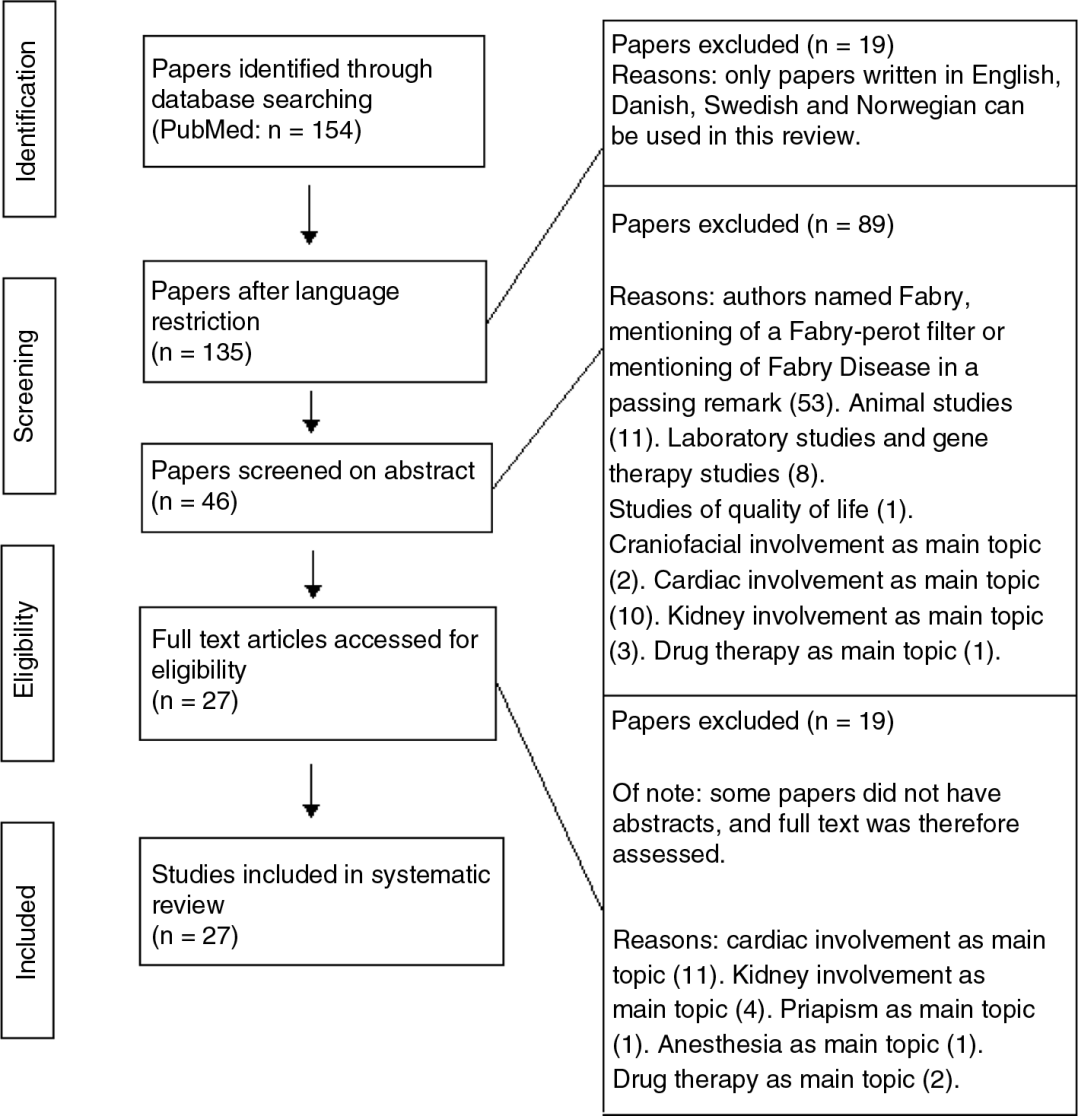
Flow chart illustrating the systematic literature search.

Of 135 papers, 108 were excluded due to the above-mentioned criteria, whereas the remaining 27 articles were relevant and have been used in this review. None of the 27 papers were written in a Nordic language. Figure 2 illustrates the literature search.

## Results

### Respiratory symptoms

The majority of the literature describes symptoms and signs of pulmonary involvement such as dyspnoea, wheezing, and dry cough in Fabry disease (5–9).

Also intermittent chest tightness, pneumothorax (7, 9, 10), haemoptysis (11, 12), recurrent pulmonary infections (13), pulmonary thromboembolism (14, 15), pulmonary infarction (11, 14) and shortness of breath and acral cyanosis upon minimal exertion (6, 8) have been described in a few studies.

A sleep study showed that 5 out of 22 patients with Fabry disease experienced Cheyne-Stokes respiration during sleep with no significant correlation to heart failure but a possible association with structural cerebral damage. Excessive daytime sleepiness in a patient with Fabry disease has also been described (16).

A study by Brown et al. (9) found 25 patients with dyspnoea, wheezing, and/or cough without simultaneous cardiac problems. Also, the study did not find any patients with symptoms under the age of 26 in the group of 25 patients (*p*<0.05).

### Pulmonary function test

A greater part of the literature described patients with an obstructive airflow limitation defined by forced expiratory volume in 1 sec/forced vital capacity (FEV1/FVC) <70% (4, 7, 8, 17–23). Furthermore, a review of Franzen et al. mentioned that obstructive lung disease was up to 10 times more frequent in patients with Fabry disease compared to the background population (24). Moreover, evidence has suggested that the pulmonary obstruction is age-dependent and increasing with age (9, 20).

Magage et al. (19) defined obstruction of the small airways as only FEV1>80% of that predicted and forced expiratory flow at 25–75% of the FVC curve (%FEF25–75)<70% of that predicted. Several patients met these criteria (19, 21, 22). The study showed that the values of %FEF25–75 decreased with the same speed in males and females and in younger and older patients. Spirometry results of Wang et al. (4) showed no correlations between increasing age and worsening of %FEF25–75.

There has been no significant responsiveness towards methacholine challenge testing (9) in patients with Fabry disease, and reversibility test with beta2-agonist was not significant (9, 20).

A few cases focusing on spirometry results of patients with Fabry disease showed restrictive ventilatory impairment and/or a mixture of restrictive and obstructive airflow limitation in patients with Fabry disease (5, 9, 21, 25).

A case report from 1961 described spirometry results suggesting mild emphysema (11).

A study by Koskenvou et al. (26) of 17 patients showed a significant association between dyspnoea and low ejection fraction (*p*<0.001) and a long QRS interval (*p*=0.04), while the symptoms did not have any association with the pulmonary function tests.

The study by Brown et al. (9) showed that dyspnoeic patients were significantly more likely to exhibit obstructive ventilatory impairment (*p<*0.005), but there was no correlation between cough and obstruction (*p*=0.10).

One study of 39 patients with Fabry disease found a significantly lower% expected FVC in males than in females (*p*=0.04) (22).

In a cohort of 15 patients undergoing a cardiopulmonary exercise test, 13 of them achieved maximal oxygen consumption (V_O2 max_) less than the predicted range (21). Other studies (4, 22) also showed patients with abnormal V_O2 max_.

Several studies described reduced gas exchange (DL_CO_) (4, 5, 7, 8, 10, 26), whereas one study (21) did not find any abnormality in a cohort of 15 patients, and others found normal diffusion capacity (9, 26). Furthermore, a ventilation–perfusion study revealed a ventilator perfusion mismatch in a 38-year-old non-smoking female patient (8).

### Tobacco consumption

In seven patients with Fabry disease, airway obstruction was worse in patients who smoked compared to non-smoking patients, but the difference was estimated as greater than what could be explained by smoking alone (10).

The study by Brown et al. (9) with 25 Fabry patients showed that smokers, ex-smokers and never-smokers had similar frequency of respiratory symptoms (cough, *p*=0.19; wheezing, *p*=0.19; dyspnoea, *p*=0.18; combination of two or more symptoms, *p*=0.12).

There was no significant correlation between obstructive lung disease, severity of obstruction, and smoking history (Brown et al., *p*=0.062) (9, 20, 22). Additionally, smokers with obstruction were not younger than non-smokers with obstruction (*p*=0.86) (9).

### Radiology

Chest X-ray and CT scan of a few patients showed patchy ground-glass pulmonary infiltrations (4, 5, 8, 26), fibrosis (19), and air trapping (8). Others showed no changes (6, 9).

Koskenvou et al. (26) found no correlation between fibrosis (diagnosed by high-resolution CT) and lung spirometry.

### Histology

Electron microscopy of induced sputum and of lung biopsy showed ‘myeloid-like’ inclusions in ciliated cells (18); and lamellar inclusion bodies (Zebra bodies) in the cytoplasm of ciliated bronchial epithelial cells (6, 10), in bronchiolar/arteriolar smooth muscle (4, 5), in endothelia of capillaries (5) and arterioles (4), veins and arteries (27), as well as in smooth muscle cells of the arteries (5, 27).

Biopsies also showed peribronchiolar fibrosis (4) and smooth-muscle hyperplasia (5, 27).

Studies hypothesise that obstructive lung disease might be due to a constriction of airways caused by accumulation of glycosphingolipids in bronchial cells (24), smooth muscle (5), fibrosis, or hyperplasia of epithelia. Rosenberg et al. (10) suggest that obstruction may be due to loss of elastic recoil.

### Enzyme replacement therapy

Infusion of agalsidase beta (1 mg/kg) biweekly has shown some effect on symptoms and pulmonary function test (8). However, one case report (5) described worsening of total lung capacity in pulmonary function tests, and worsening of fibrosis found on CT and X-ray 49 months into treatment. It was, therefore, suggested that the progression of fibrosis cannot be reversed if a certain ‘point of no return’ is reached.

An interventional study of six patients receiving either ERT or placebo showed an increased exercise tolerance in patients receiving ERT (21). Tables (1– 5) summarize the results of the included studies and case reports.

**Table 1:** A summary of the cross-sectional studies concerning lung involvement in Fabry disease

Ref. no. and year of publ.	No. of patients	Methods	Outcome	Conclusion
(3) 2010	90 probands	Genetic analysis, spirometry, ECG, echocardiography	Three were found to carry a *GLA* missense mutation. These patients experienced airflow obstruction, reduced lung diffusion capacity, ventricular tachycardia, and atrial fibrillation	Enzyme measurements were sufficient in men, whereas genetic testing was needed in women
(4) 2007	44 females	Questionnaire on symptoms, pain, and quality of daily living, cerebral MRI, ECG, spirometry, biochemistry, non-invasive exercise test	Quality of life was reduced, pain affected mood and enjoyment of life. Manifestations were present above that predicted for random X-inactivation of the normal allele	Heterozygote female carriers experienced a broad variety of symptoms and reduced quality of life. The different phenotypes in women might be partially explained by variable X-inactivation
(9) 2008	25 males	Questionnaire on smoking history, methacholine challenge testing, spirometry, biochemistry, X-ray, radionuclide scan	36% experienced dyspnoea and 24% experienced cough and/or wheezing. Nine had airway obstruction which was associated with age, dyspnoea, and wheezing. No association between airway obstruction and smoking history	Airway obstruction increased with age and occurred regardless of smoking history
(10) 2000	6 males, 1 female	Biochemistry, spirometry, ventilation and perfusion scans, X-ray, bronchoscopy, questionnaire on smoking history	All patients had obstruction to airflow, worse in patients who smoked. Airway epithelial cells contained inclusion bodies	Mild smoking aggravated airflow obstruction
(16) 2013	12 males, 11 females	Cerebral MRI, electro-neurography, echocardiography, polysomnography	Five out of 22 patients had central sleep apnoea with Cheyne-Stokes respiration. There were widespread structural changes in patients with Fabry disease when compared to the healthy controls	Significant association between severity of Cheyne-Stokes respiration and microstructural changes within the brainstem. The changes in the brainstem might correlate with central sleep apnoea with Cheyne-Stokes respiration in patients at risk of white matter lesions
(20) 2006	67 (from the Fabry Outcome Survey)	Spirometry, questionnaire on smoking history	34% had airway obstruction	34% had airway obstruction. Unknown whether smoking was involved
(22) 2005	15 males, 24 females	ECG, spirometry, echocardiography, non-invasive cardiopulmonary exercise test	46% exhibited a significant decrease in diastolic blood pressure during exercise. The drop was evident in 9 of the 24 female patients	The significant decrease in diastolic blood pressure in patients with Fabry disease may explain deficits in exercise tolerance
(26) 2010	6 males, 11 females	Spirometry, pulmonary HRCT, bicycle stress test, ECG, cardiac MRI, questionnaire on lifestyle and symptoms	LVH, reduced exercise capacity, normal ECG parameters apart from changes related to LVH, mild reduction in vital capacity and forced expiratory volume in 1 sec, mean values in diffusion capacity test within normal limits	LVH and reduced exercise capacity were the most apparent cardiopulmonary changes but had little association to cardiopulmonary symptoms

All studies included physical examination and interview about past medical history. *GLA*, α-galactosidase A gene; HRCT, high-resolution CT; LVH, left ventricular hypertrophy.

**Table 2:** A summary of the longitudinal study concerning lung involvement in Fabry disease

Ref. no. and year of publ.	No. of patients	Methods	Outcome	Conclusion
(19) 2007	50 (39 were follow-ups)	Spirometry, bronchodilatory test	Reduction in spirometric parameters corresponding to airway obstruction. Age-dependent reduction of %FVC and %FEV1 in men	Age- and sex-dependent progression of pulmonary involvement

FVC, forced vital capacity; FEV1, forced expiratory volume in 1 sec.

**Table 3:** A summary of the interventional study concerning lung involvement in Fabry disease

Ref. no. and year of publ.	No. of patients	Methods	Outcome	Conclusion
(21) 2006	19 (6 in RCT)	Cardiopulmonary exercise test, ECG, spirometry, physical examination	Four patients had airway obstruction, two had decreased FVC. None had a diffusing capacity less than 75%. Reduction in exercise capacity	Exercise tolerance increased in patients receiving ERT

RCT, randomised controlled trial; FVC, forced vital capacity; ERT, enzyme replacement therapy.

**Table 4:** A summary of the longitudinal case reports concerning lung involvement in Fabry disease

Ref. no. and year of publ.	No. of patients	Methods	Outcome	Conclusion
(5) 2008	One female	Spirometry, biochemistry, X-ray, CT scan, open lung biopsy, initiation of ERT	Mixed restrictive/obstructive pattern, patchy ground-glass infiltrations, peribronchiolar fibrosis, and smooth-muscle cell hyperplasia. Inclusion bodies. ERT stabilised the airway obstruction	Pulmonary involvement is due to lysosomal storage on cells. Treatment with ERT was able to stabilise the airway obstruction
(8) 2007	One female	Biochemistry, spirometry, ventilation-perfusion scan, echocardiography, CT scan. Initiation of ERT	LVH, reduced diffusion capacity, a combination of ground-glass infiltrations and air trapping. Improvement of pulmonary signs but persisting opacities and air trapping after ERT	ERT might improve pulmonary signs but has no effect on ground-glass infiltrations and air trapping found on CT scan
(18) 2013	One female	Spirometry, renal biopsy, lung biopsy, biochemistry, electron microscopy	Patient with the diagnosis of COPD received a lung transplant. Later, Fabry disease was diagnosed	Rare differential diagnosis might be hidden under more common diseases. Respiratory impairment cannot be denied

All studies included physical examination and interview about past medical history. ERT, enzyme replacement therapy; LVH, left ventricular hypertrophy; COPD, chronic obstructive pulmonary disease.

**Table 5:** A summary of the cross-sectional case reports concerning lung involvement in Fabry disease

Ref. no. and year of publ.	No. of patients	Methods	Outcome	Conclusion
(6) 2000	One female	X-ray, spirometry, examination of induced sputum	Mild-to-moderate chronic airway limitation, accumulation of lamellar inclusion bodies	Examination of induced sputum is clinically useful in patients with known Fabry disease
(7) 1978	One male	Biochemistry, skin biopsy, spirometry	Angiokeratomas, pulmonary involvement	Significant primary pulmonary involvement and skin lesions in an early stage of Fabry disease
(11) 1961	One male	ECG, X-ray, biochemistry, spirometry, skin biopsy, renal biopsy	Mild emphysema, pulmonary infarcts, angiokeratomas, thickening of Bowman's capsule, myocardial damage	Widespread system involvement
(12) 2000	One male	ECG, bronchoscopy, electron microscopy, autopsy	Pulmonary haemorrhage. Cholesterol clefts within the renal arterioles and lamellar inclusion bodies	Rare case of Fabry disease coexisting with cholesterol crystal embolisation
(13) 2004	One male	Mutation analysis	Nine-year-old boy presented with fever, acroparaesthesia, respiratory infection	The patient was diagnosed with Fabry disease
(14) 2007	One male	X-ray, brain CT, light-microscopic histochemistry, electron microscopy, autopsy	An acromegaly-like condition. Pulmonary thromboembolism, cerebral infarcts. Lipid deposits observed in endothelial cells and smooth muscle	Lipid deposits causing severe organ failure
(15) 1990	One male	ECG, echocardiography, electron microscopy, autopsy	Pulmonary thromboembolism, LVH, lipid storage solely in cardiocytes	Rare monosymptomatic case, representing a new variant of Fabry disease
(25) 1972	Three males	X-ray, pulmonary scintigraphy, spirometry, smoking history, biochemistry	No abnormalities on X-ray, heart failure in one patient. No clear pattern of symptoms and signs	No evidence of primary pulmonary involvement. Might be related to other factors
(17) 2007	One male	ECG, biochemistry, ultrasonography of kidneys and urinary tract, renal scintigraphy, spirometry	Systemic involvement. Myocardial infarction, LVH, COPD, angiokeratomas	Myocardial infarction, LVH, COPD, angiokeratomas. ERT initiated
(23) 2008	Two males	3-lead ECG and spirometry in connection with anaesthesia for a kidney transplant	Standard anaesthesiological protocol resulted in uneventful awakening after uncomplicated surgery	Patients with Fabry disease need a careful preoperative evaluation of cardiopulmonary functionality and advanced haemodynamic monitoring during surgery
(27) 1991	One male	Autopsy, electron microscopy	Inclusions within pulmonary arteries, arterioles, veins, and alveolar walls (zebra bodies)	Controversy whether accumulations of glycolipids in the lung affected respiratory function

All studies included physical examination and interview about past medical history. LVH, left ventricular hypertrophy; COPD, chronic obstructive pulmonary disease; ERT, enzyme replacement therapy.

## Discussion

There is limited consensus regarding lung manifestations among patients suffering from Fabry disease. Few publications have investigated lung involvement in patients with Fabry disease, partly because Fabry disease is a rare disease, which has resulted in studies of small cohorts. Also, none of the publications mentioned in this review have evaluated the power of their respective studies. Therefore, there is a great risk of type-2 errors. Larger multicentre surveys would certainly improve our knowledge in this area. Moreover, the majority of the publications mentioned in this review were case reports, also with a low level of evidence.

The majority of the literature agreed that the following respiratory symptoms might develop: dyspnoea, wheezing, and dry cough. These findings were found independently of airflow limitation.

There are divergent opinions regarding whether the symptoms are caused by pulmonary involvement or heart disease (9, 25, 26).

Pulmonary function tests of patients with Fabry disease also showed different results. The majority of the studies and case reports showed obstructive airflow limitation, but emphysema, restrictive ventilatory impairment, and/or a mixture of restrictive and obstructive airflow limitation have also been described. However, one should be aware that the definition of obstructive, restrictive, and mixed airflow limitation was not homogenous throughout the literature, and not all of the articles have mentioned the individual spirometry values. In this review, obstruction of airways is defined as FEV1/FVC<70%, and as the patients with Fabry disease are middle-aged patients, this seems to be acceptable and not leading to either over- or under-estimation of chronic obstructive pulmonary disease (COPD).

There were no signs of variable airway disease such as those found in patients with asthma, as no airway hyperresponsiveness (9) or reversibility towards beta2-agonist was found (9, 20). This argues against airway inflammation generally found in Th2 diseases such as asthma in Fabry disease. However, there is a need for further studies with a greater statistical power to decline beta2-agonist as a way of treatment in Fabry disease.

The presence of small airway disease among patients with Fabry disease was found in several studies (19, 21, 22). The study of Magage et al. showed that the values of %FEF25–75 decreased with the same speed in males and females and in younger and older patients, which could indicate that an airway obstruction starts in the peripheral airways, and along with the progression of Fabry disease, the larger airways gradually become affected (19).

In their study population of 25 with respiratory symptoms, Brown et al. reported no patients under the age of 26, and only one article in the remaining literature in this review has reported a young patient with lung involvement (9-year-old with recurrent respiratory infections). The suggestion that pulmonary involvement is age-dependent and increasing with age is, therefore, strengthened.

On the other hand, the onset of lung involvement in Fabry disease (the age with which the symptoms occur or whether or not they occur at all) might be dependent on phenotype. To date, no study comparing respiratory symptoms and pulmonary function test with phenotypes has been performed.

Development of emphysema or lung fibrosis with reduced gas exchange (DLco) has only been found in a few cases (4, 5, 7, 8), which indicated limited statistical support for destruction of the lung parenchyma in patients with Fabry disease. Lung involvement in patients with Fabry disease might occur because of constriction of airways caused by smooth-muscle hyperplasia or accumulation of glycosphingolipids in bronchial cells. Rosenberg et al. (10) suggested that obstruction may be due to loss of elastic recoil, but up till now no study has been able to find the mechanisms behind lung involvement in Fabry disease.

It is well known that tobacco consumption is associated with the development of respiratory symptoms and COPD, but this review did not find support for increased effects of smoking on the development of lung symptoms in patients with Fabry disease. There seems to be no relationship between smoking and the degree of obstruction (9, 20, 22), and smokers with obstruction were not younger than non-smokers with obstruction (*p*=0.86) (9). This lack of association might be a question of statistical power in the different studies.

ERT is used in patients with other organ manifestations, such as kidney failure and LVH, and has also been evaluated in a few patients with Fabry disease with lung involvement. ERT might reduce or stabilise symptoms and lung manifestations (5, 8), but the current literature is not convincing. It was suggested (5) that the progression of fibrosis cannot be reversed if a certain ‘point of no return’ is reached.

The current patients receiving ERT had lived with Fabry disease for decades before treatment was initiated. If the hypothesis about a ‘point of no return’ where fibrosis, or maybe other manifestations, cannot be reversed, clinicians should consider the benefits of initiating treatment early in the disease development.

## Conclusion

Examination of existing literature concerning lung involvement in patients with Fabry disease shows little consensus. Predominantly, the literature reports of patients having dyspnoea, wheezing, dry cough, obstructive airway limitation, and histological abnormalities such as lamellar inclusion bodies in epithelial cells and smooth-muscle hyperplasia. Smoking has not been significantly associated with increased obstructive airway limitation or respiratory symptoms. It is still debated whether or not cardiac involvement causes respiratory symptoms seen in patients with Fabry disease.

ERT has shown improvement in some aspects of lung involvement.

There is a need for further studies investigating lung involvement, progression, effects of smoking, and effects of ERT as well as treatment with beta2-agonists in patients with Fabry disease.
